# Bilateral Maxillo-Mandibular Syngnathia in a Newborn

**Published:** 2014-10-20

**Authors:** A. El Madi, K. Khattala, M. Rami, Y. Bouabdallah

**Affiliations:** Department of Pediatric Surgery, Hassan II University Hospital; Fes: 30000 Morocco

A 5-day-old newborn, product of non-consanguineous marriage, presented with inability to completely open oral cavity and feeding difficulty. On examination, there was a good sucking reflex and limited mouth opening (Fig. A) with retrognathia (Fig. B). Maxillary CT with 3D reconstruction revealed hypoplasia of the ascending branches of the mandible; the temporomandibular joints were normal (Fig. C, D) with bilateral Maxillomandibular Syngnathia (Fig. E, F).

**Figure:**
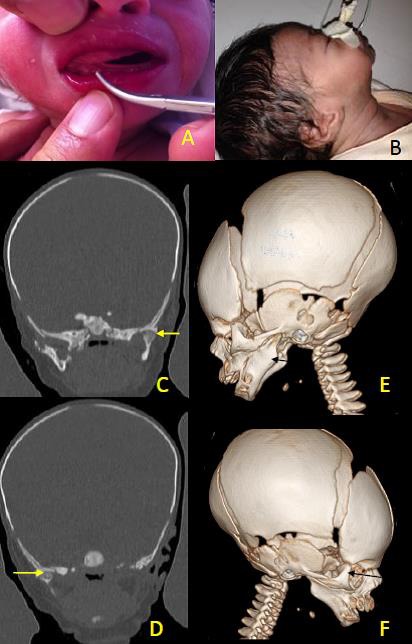
Figure 1 : Clinical and radiological demonstration.

Congenital fusion of the maxilla and mandible is a rare anomaly which is usually diagnosed at birth owing to inability to open mouth and feed. Congenital synostosis of the mandible and maxilla is even less common than synechiae, with only 25 cases reported in the literature [1]. Congenital maxillomandibular fusion may be associated with other anomalies such as Van der Woude syndrome, or aglossia-adactylia syndrome [2]. Dawson et al [3] divided bony syngnathia into two broad categories: Type 1 - simple syngnathia without any associated congenital anomalies of head and neck and Type 2 - complex syngnathia with associated congenital anomalies of head and neck. This is further sub classified into Type 2a with associated aglossia and Type 2b with associated agenesis or hypoplasia of proximal mandible [3]. No consensus on management is there given the rarity of the disease and anesthesia is difficult in these patients.

## Footnotes

**Source of Support:** Nil

**Conflict of Interest:** Nil

